# Benign splenosis mimicking peritoneal seeding in a bladder cancer patient: a case report

**DOI:** 10.4076/1757-1626-2-8982

**Published:** 2009-09-11

**Authors:** Stefania Rizzo, Lorenzo Monfardini, Maddalena Belmonte, Bernardo Rocco, Massimo Bellomi

**Affiliations:** 1 Department of Radiology, European Institute of Oncology, via Ripamonti 435, Milan, 20141, Italy; 2 School of Medicine, University of Milan, via Festa del Perdono 7, Milan, 20122, Italy; 3 Department of Urology, European Institute of Oncology, Via Ripamonti 435, Milan, 20141, Italy

## Abstract

**Introduction:**

Splenosis is a post-traumatic autotrasplantation and proliferation of splenic tissue in ectopic sites. These implants may mimic malignancy in healthy patients or peritoneal metastases in cancer patients. When a previous history of splenic injury is known, the finding of soft tissue nodules in many thoracic and abdominal locations might raise the suspicion of the benign condition of splenosis, in order to avoid unnecessary surgery or chemotherapy.

**Case presentation:**

A 56-year-old man with history of persistent hematuria from bladder cancer was referred to our Institution for suspected peritoneal carcinosis. For staging purposes he underwent abdominal computed tomography and ultrasound. The integration of patient's history and imaging results led to the diagnosis of peritoneal splenosis. The patient therefore underwent regular Trans Urethral Resection of Bladder for the known malignancy; while no treatment was necessary for splenosis. Two years follow-up was negative for metastases.

**Conclusion:**

Splenosis is a benign condition after traumatic splenectomy which should be taken into account in the differential diagnosis with peritoneal seeding of malignancy because its appearance may resemble malignancy.

## Introduction

Traumatic disruption of the splenic capsule causes fragments of splenic tissue to be seeded throughout the peritoneal cavity [1]. It has been demonstrated that splenic implants can survive and grow when transplanted to ectopic sites, such as the splenic fossa, any site of the peritoneal cavity, gastro-intestinal tract, liver, kidney, thorax, subcutaneous tissues and even in the head [2].

Since these implants may mimic malignancy or peritoneal metastases, in cancer patients with previous history of splenic injury, the finding of soft tissue nodules in the abovementioned locations might raise the suspicion of splenosis in order to avoid unnecessary surgery or chemotherapy.

This report describes the case of a bladder cancer patient with peritoneal splenosis mimicking metastases.

## Case presentation

A 56-year-old white man native from Italy, was admitted to our Institution for a diagnosis of bladder cancer and a previous CT scan suggesting peritoneal carcinosis. His history started about 4 months in advance for the occurrence of hematuria. His previous personal history was unremarkable, other than a car accident about 20 years in advance, which requested a long hospital staying and a splenectomy.

At our hospital he underwent a Trans-Urethral Resection of Bladder (TURB) which led to the diagnosis of spinocellular carcinoma of the bladder. Usual patient's blood tests, including complete blood cell count and tumour markers, were unremarkable.

**Figure 1:**
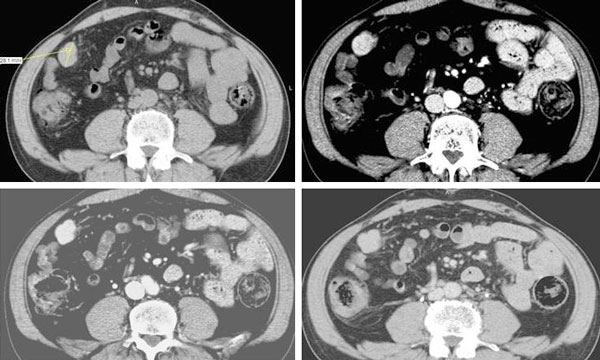
**Abdominal CT scan of a splenic nodule before and after contrast medium injection**. This 28 mm peritoneal nodule located in the right paracolic space showed slight hypodensity during the pre-contrast phase, **(a)** inhomogeneous contrast-enhancement during the arterial phase **(b),** slightly iso-hypodenity during the portal **(c)** and equilibrium phase **(d).**

The patient underwent a second computed tomography (CT) scan for staging purposes, showing the presence of many peritoneal hypodense masses, with heterogeneous contrast-enhancement during the arterial phase and almost isodense to the hepatic parenchyma during both the portal and equilibrium phases (Figure 1).

**Figure 2:**
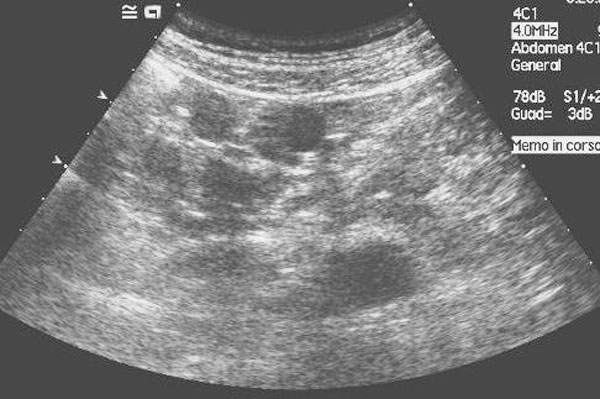
**Second-look ultrasound of the nodule in figure **1. US shows homogeneous splenic-like echogenicity of the nodule in the paracolic space, thus confirming its splenic origin.

The spleen was not visualized on the CT scan and splenosis was thereafter suspected as a possible diagnosis and a second-look ultrasound (US) exam confirmed the presence of peritoneal solid splenic-like masses on the sites indicated by CT (Figure 2).

Diagnosis of post-splenectomy splenosis was confirmed and the patient underwent usual TURV as treatment for bladder cancer, with no chemotherapy, or further treatment for splenosis.

## Discussion

Splenosis is a relatively common finding in clinically silent patients, frequently misdiagnosed because of the lack of symptoms. According to Normand et al it involves 16% to 67% of patients with past splenic trauma and/or past splenectomy [3]. On the other hand, Khosravi et al found only five cases of splenosis in a retrospective review of a 29-year period of the pathology registry [1], while Lin et al identified eight patients with splenosis in a period of 8 years [2].

Based on the location of the splenic nodules, differential diagnoses to be considered may be endometriosis, in presence of pelvic implants [4], peritoneal mesothelioma in case of peritoneal seeding [5], renal cancer [6] in case of renal implants, abdominal lymphomas in case of retroperitoneal locations mimicking lymph nodes [7], hepatic adenomas in case of intra-hepatic implants [8] and peritoneal metastases.

Thus, patient's history is substantial; secondarily imaging modalities to consider are: CT, US, magnetic resonance (MR), scintigraphy.

If the splenic implant is intra-hepatic, CT imaging may show hypodense masses with strong enhancement at the early phase and pooling enhancement at delayed phase [9]. Grande et al described multiple well-demarcated nodules without calcifications, slightly hypodense compared to the liver at non-contrast CT, showing as lobular or oval well-circumscribed structures, hyperdense in the arterial phase and isodense in the portal phase after contrast administration [10]. Accordingly, Imbriaco described a well-demarcated intra-hepatic 3 cm mass, hypodense compared with the surrounding liver parenchyma at unenhanced CT, heterogeneously enhanced in the arterial phase, becoming hypodense during the portal end equilibrium phases [5].

In our case study, according to previous studies, CT scan showed multiple hypodense peritoneal masses with a maximum diameter of 15-20 mm, which characteristically had slightly hypodensity in the pre-contrast phase, inhomogeneous contrast-enhancement during the arterial phase and iso - slightly hypodensity to the liver parenchyma during the portal and equilibrium phase.

Ultrasound may incidentally show hypoechoic masses on the surface of solid abdominal organs, such as the liver [9] or the kidney [5]. It may also show multiple solid masses with a smooth round or ovoid shape, with a homogeneous echo-texture and a hyperehoic peripheral rim, without specific arterial or venous Doppler signals [10]. In our case, a second-look ultrasound, confirmed the presence of multiple solid nodules with homogeneous splenic-like echogenicity in the splenic fossa and in the peritoneum.

MRI may be considered as an alternative modality for the identification of splenosis, in case of uncertainty of diagnosis with other exams. Splenic implants have been described as hypointense on T1-weighted images and hyperintense on T2-wieghted images, therefore similar to normal splenic tissue [11,12].

Nowadays scintigraphy (performed with heat-demaged 99Tc-labelled red blood cells) is still considered the more sensitive and specific imaging modality for the diagnosis of splenosis [10,13,14], being sensitive even in early splenosis, in cases where splenic tissue is minimally present, in functional hyposplenism and in case of poor splenic uptake. This because splenic tissue takes up more than 90% of damaged red blood cells [15].

Once splenosis is diagnosed with anyone of the previous modalities, no more treatment is usually required unless the patient is symptomatic.

## Conclusion

In conclusion this report describes a case of a bladder cancer patient with peritoneal lesions related to personal history of post-trauma splenectomy (splenosis). Since this benign condition may mimic metastases, it should be kept in mind in managing cancer patients with personal history of post-traumatic splenectomy, in order to avoid unnecessary surgery or chemotherapy.

## Consent

Written informed consent was obtained from the patient for publication of this case report. A copy of the written informed consent is available for review by the Editor-in-Chief of this journal.

## Competing interests

The authors declare that they have no competing interests.

## Authors' contributions

BR analyzed and interpreted the patient data regarding the oncological disease and the requested treatment. SR performed the radiological examinations (CT and ultrasound) of the abdomen and was the major contributor in writing the manuscript. LM and MB contributed to the literature search. MB supervised the entire work. All authors read and approved the final manuscript.
